# Pathogens in Ornamental Waters: A Pilot Study

**DOI:** 10.3390/ijerph13020216

**Published:** 2016-02-15

**Authors:** Maria Nascimento, Joao Carlos Rodrigues, Lucia Reis, Isabel Nogueira, Patricia A. Carvalho, João Brandão, Aida Duarte, Luisa Jordao

**Affiliations:** 1 Department of Environmental Health (DSA), National Institute of Health Dr Ricardo Jorge (INSA), Avenida Padre Cruz, Lisboa 1649-016, Portugal; maria_d_n@hotmail.com (M.N.); joao.brandao@insa.min-saude.pt (J.B.); 2 Department of Infectious Diseases (DDI), INSA, Avenida Padre Cruz, Lisboa 1649-016, Portugal; joao.rodrigues@insa.min-saude.pt (J.C.R); lucia.reis@insa.min-saude.pt (L.R.); 3 Departament of Chemical Engineering, Instituto Superior Técnico, University of Lisboa (UL), Av Rovisco Pais, Lisboa 1049-001, Portugal; isabel.nogueira@ist.utl.pt (I.N.); pac@ist.utl.pt (P.A.C.); 4 Faculty of Farmacy, Departament of Microbiology and Imunology; iMed.UL, UL, Av Prof Gama Pinto, Lisboa 1649-003, Portugal

**Keywords:** waterborne pathogens, children’s health, play behavior, environmental exposures, biofilms

## Abstract

In parks, ornamental waters of easy access and populated with animals are quite attractive to children and yet might hide threats to human health. The present work focuses on the microbiota of the ornamental waters of a Lisboa park, characterized during 2015. The results show a dynamic microbiota integrating human pathogens such as *Klebsiella pneumoniae*, *Aeromonas* spp. and *Enterobacter* spp., and also antibiotic resistant bacteria. *K. pneumoniae* and *Aeromonas* spp. were present as planktonic and biofilm organized bacteria. *In vitro K. pneumoniae* and *Aeromonas* spp. showed an enhanced ability to assemble biofilm at 25 °C than at 37 °C. Bacteria recovered from biofilm samples showed an increased antibiotic resistance compared to the respective planktonic counterparts.

## 1. Introduction

Life in metropolitan areas is as much convenient as it can be stressful. In order to decompress there is a growing tendency to enjoy the free time in city parks, practicing open air sports or simply interacting with nature. City planners have included parks and green areas in urban environment since always. These areas are beneficial for public health and environmental preservation. Among the contribution for public health is the physical activity with impact on the psychological condition and social interactions [1]. Evidence for the beneficial impact on children cognitive development resulting of the interaction with green areas has also been provided [2].

Artificial lakes, ornamental fountains and other water courses are present in the majority of city parks and green areas. Water is essential for life and promotes the general well-being. Nevertheless, the presence of infectious agents, toxic chemicals, and radiological hazards could represent a danger for human health. For instance decorative fountains have been related to outbreaks of the Legionnaire’s disease [3]. Another source of bacteriological contamination lies in bacteria carried by animals that inhabit the lakes. Several studies have provided evidence for the presence of potential human pathogens in the water containing ornamental fish [4,5]. The introduction of non-indigenous species of fish and other animals is a major threat to global diversity, natural habitats and also for the human health [6]. Non-indigenous species can work as either carriers or vectors of infectious agents. This is especially true when the potential invader may act as keystone species or is a known disease carrier. American crayfish species are a vector of the pathogenic fungus *Aphanomyces astaci* (Oomycetes) responsible for the crayfish plague fatal for indigenous European, Japanese and Australian freshwater crayfish species that have been found to be highly susceptible to fungus species [7]. In Germany, crayfish were introduced as aquarium pets, illustrating well the major burden caused on biodiversity after direct habitat destruction by either accidental or deliberate introduction of exotic species [7]. *Salmonella* spp. are an example of human pathogens responsible by gastrointestinal disorders [8] and meningitis [9] and are carried by exotic species such as reptiles (e.g., lizards) and amphibians (e.g., turtles). *Salmonella* infections can result from contact with these animals or the environment in which they lived, including the water from containers oraquariums [10]. 

The majority of environmental microorganisms live associated with a matrix within a structure known as a biofilm [11]. Biofilm-embedded microorganisms are more resistant to pH and temperature changes, nutrient deprivation and other stress factors [11,12]. Microorganisms carried by exotic species are more likely to be exposed to non-physiological conditions (stress factors) which might trigger biofilm assembly in order to survive in non-native habitats. The link between bacterial biofilm assembly and emergence of antibiotic resistance, together with the increased use of antibiotics in animal and plant health, raises several concerns for public health [13].

Behavioral epidemiology has shown that individual behavior is a key factor of infectiontrajectories [14]. The curious nature of children, always available to closely interact with the surrounding environment—especially with colorful moving entities, such as ducks and fish—together with their immunity peculiarities, suggests an increased vulnerability to infection by waterborne bacteria. For these reasons the aim of this pilot study was to characterize the microbiota from different ornamental lakes and fountains of a park located in Lisboa, Portugal. A special attention was given to the ability of environmental bacteria to persist within biofilms and the antibiotic susceptibility profiles of clinically relevant members of the ornamental waters microbiota.

## 2. Experimental Section 

### 2.1. Collection of Samples

Waters samples were collected within a park with a total area of 26 hectares, located in the northern area of Lisboa, Portugal. The park has children playgrounds surrounded by an extensive lawn and wooded areas, as well as two lakes and two ornamental fountains with different characteristics. One of the lakes (L1) houses a duck population and has easily accessible margins, whereas the other lake (L2) is deeper, does not have a resident animal population and its margins are of difficult access. One of the ornamental fountain (L3) has reduced dimensions, whereas the second (L4) in addition to the bigger dimensions, has a stationary and dynamic water filling during winter and summer, respectively. From L1, L3 and L4 one sample of 500 mL surface water was collected by shore. Due to L2 topology surface water was collected by shore from the center of the lake using a van Dorm apparatus. Three biofilm samples from L1 and L4 cement borders were also collected with a swab on a surface area of 10 cm^2^ and introduced in 10 mL phosphate buffer saline (PBS). All samples were transported in the dark and stored at 5 °C until further process. This storage step was never longer than 24 h. The first samples were collected during February 2015 from L1 to L4 being repeated for L4 during May 2015.

### 2.2. Isolation and Identification of Bacteria 

Water samples were homogenized by inverting the recipient several times and biofilm swabs solution were vortexed to ensure bacterial release prior to filtration through membranes filters 0.45 μm (Merck-Millipore, Darmstadt, Germany) using a filtration slant (Merck-Millipore). In all cases 10 mL of the sample were filtrated. The membranes were then transferred either to non-selective or selective solid culture media and incubated at three different temperatures (30 °C, 37 °C and 44 °C) either for 24 h to 48 h (bacteria and yeast) or 5 days (fungi). Non selective media was Plate Count Agar (PCA) and among selective media Sabouraud was used for yeast and fungi, Drigalsky, Cystine Lactose Electrolyte Deficient (CLED) and Violet Red Bile Agar (VRBA) for Gram-negative bacteria, Cetrimide and Manitol salt agar for *Pseudomonas* spp. and *Staphylococcus* spp. respectively. All culture media were purchased from bioMerieux (Marcy l’Etoile, France). Microbial identification was performed using API20E for Gram negative bacteria and VITEK 2 systems for fastidious bacteria with appropriate cards (bioMerieux). Briefly, a homogeneous microbial suspension was prepared from over-night cultures in 0.45% sodium chloride solution adjusted to a turbidity of 0.5 McFarland (~1.5 × 10^8^ colony-forming units (CFU)/mL). The microbial suspension and the identification card selected according to the oxygen use pattern, Gram staining and morphology were loaded into API20E (bioMérieux) or VITEK 2 (bioMérieux) apparatus and further processed according to the manufactures indications.

### 2.3. Antimicrobial Susceptibility Testing

The antimicrobial activity was tested using the disk diffusion method described by EUCAST guidelines [15] or by microdilution broth method (Vitek 2 system) using the following antibiotics: amikacin, amoxicillin, amoxicillin/clavulanic acid, ampicillin, ampicillin/sulbactam, cefepime, cefotaxime cefoxitin, ceftazidime, ciprofloxacin, ertapenem, fosfomycin, gentamicin, levofloxacin, meropenem, nitrofurantoin, piperacillin/tazobactam and trimethoprim/sulfamethoxazole. Bacterial suspension adjusted to a turbidity of 0.5 McFarland (~1.5 × 10^8^ (CFU)/ml) were diluted in 0.45% sodium chloride solution (145 μL bacterial suspension/ 3 mL 0.45% NaCl) and loaded into the Vitek 2 apparatus with an appropriate card and further processed according to the manufacturer’s instructions. The results were interpreted according to EUCAST guidelines [15].

### 2.4. Biofilm Assay

The assay was performed in triplicate using 96-well flat-bottomed cell culture plates (Nunc, New York, NY, USA) as described previously [16]. Briefly, bacterial suspensions at a final concentration of 10^7^ (CFU)/mL were prepared in 0.9% sodium chloride from overnight cultures in Mueller Hinton (MH) agar and ten-fold diluted in MH broth (Oxoid, Basingstoke, UK). Two-hundred microliters were distributed to each well, MH broth being used as the negative control. The plates were incubated either at 25 °C or 37 °C to allow biofilm formation for different time periods. The well content was removed, and each well was vigorously washed three times with sterile distilled water. The attached bacteria were stained for 15 min with 100 μL violet crystal at room temperature, washed with distilled water three times and allowed to dry at room temperature. The violet crystal was dissolved in 95% ethanol (Merck), and the optical density at 570 nm was read using a (SpectraMax 340PC; Molecular Devices, Sunnyvale, CA, USA).

### 2.5. Scanning Electron Microscopy (SEM)

Cement pieces with approximately 0.8 cm^2^ areas were sterilized by moist heat (120 °C for 20 min). For SEM analysis, cement pieces were placed within 24 wells cell culture plates (Nunc) and biofilms were allowed to form for 24 h at 25 °C. Samples were prepared as described before by Bandeira and colleagues [16], mounted on the sample holder with carbon tape, sputter-coated with carbon (20 nm) using a QISOT ES Sputter Coater (Quorum Technologies, Laughton, UK), and were analyzed under a JSM-7001F scanning electron microscope (JEOL, Tokyo, Japan).

## 3. Results and Discussion

### 3.1. Characterization of the Bacterial Population

The first water sampling was performed during February 2015. Fungi and yeast were absent from all water samples, but an uncountable number of colony forming units (CFU > 300/10 mL) was observed on PCA. The absence of CFU on cetrimide and mannitol salt agar excluded the presence of *Pseudomonas* spp. and *Staphylococcus* spp., respectively. The remaining media, incubated either at 37 °C or 44 °C, allowed the identification of members of the Enterobactereaceae family, such as, *Klebsiella pneumoniae*, *K. pneumoniae* ozaenae, *Enterobacter* spp., *Serratia marcescens*, *S. rubidea* and *S. odorifera*. Nonfermentative oxidase-positive bacteria *Elizabethkingia meningoseptica*, *Stenotrophomonas maltophilia* and *Vibrio metschnikovii* were also identified (Table 1). Fountains and lakes showed different bacterial species. In L1, corresponding to the lake hosting the duck population, higher microbiota diversity was found. 

The occurrence of *Klebsiella* spp. in water, an etiological agent of infectious diseases in adult and pediatric populations, both in the community and hospital with high antibiotic resistance rates raised justified concerns. For this reason we decided to evaluate the antibiotic susceptibility of all bacteria isolated from water samples. The bacterial population susceptibility profile to antibiotics is shown in Table 1. While *E. meningoseptica*, *S. maltophilia* and *V. metschnikovii* showed resistance to amoxicillin and cefoxitin, an indicator of expression of a chromosomal AmpC-type cephalosporinase. The antibiotic susceptibility pattern of *K. pneumoniae* was an unexpected result since it was resistant only to amoxicillin, an indicator of strains producing TEM or SHV-like broad spectrum *β*-lactamases classified as penicillinase.

After three months, May 2015, we verified that the ornamental fountain (L4) had a fish population composed by goldfish and Koi carp. The bacterial species identified during February were replaced by other species, namely *Aeromonas sobria*, *Bacillus* spp., and *Enterobacter aerogenes*. Another difference was the identification of fungi *Aspergillus* and *Penicillium* species and of the yeast *Rhodotorula* spp. The occurrence of *Aeromonas* could not be directly attributed to the fish introduction, although it is an interesting and concerning observation. *Aeromonas* spp. are widely distributed in the environment, inhabit an aquatic environment and also make up a part of normal intestinal microbiota of healthy fish. *Aeromonas* spp. are opportunistic and zoonotically important bacteria associated with diseases as a result of their virulence and pathogenesis. These bacteria have been isolated from either fish or fish water (transport water) are responsible for gastroenteritis in humans, mainly travel diarrhea [17,18]. Another concern raised during this analysis was the identification of *Enterobacter aerogenes*, listed among the major species responsible for acute pyelonephritis in children [19] and with high degrees of antibiotic resistance. The antibiotic susceptibility profiles for *A. sobria*, *A. veroni* and *E. aerogenes* are shown in Table 2, all *Aeromonas* species were susceptible to the antibiotics tested but *E. aerogenes* had intermediate susceptibility to nitrofurantoin, an antibiotic recommended for treatment of urinary infection. Furthermore, the later was resistant to *β*-lactam antibiotics, mainly aminopenicillins and cefoxitin, suggesting the expression of an AmpC-type cephalosporinase gene. 

### 3.2. Biofilms

Biofilm sampling from lake L1 was performed only in February since in May the lake was empty. From ornamental fountain L4 two samples were collected in February and in May after the introduction of fish. In February, from biofilm samples was identified *K. pneumoniae* at lake L1 but not at fountain L4. In contrast, in May, at fountain L4 was found a multi-microbial biofilm, composed by *A. sobria*, *A. veronii*, *Chromobacterium violaceum* and fungi. Several factors could be involved in this outcome: the atmospheric temperature increase linked with the change of seasons might have played a role, as well as interactions between different bacterial species. An antagonist relation between at least one species of the recognized member of healthy fish microbiota, *Aeromonas punctata*, and *K. pneumoniae* biofilms have been described. The production of depolymerise enzymes by *A. punctata* is responsible for disruption of *K. pneumoniae* capsule integrity causing biofilm dispersion. Although this fact has been only demonstrated for *K. pneumoniae* strains harbouring a K2 capsule, we cannot exclude the hypothesis of its occurence for other capsular phenotypes [20]. Furthermore the absence of *K. pneumoniae* after fish introduction in the fountain also supports the speculation that either the fish or their transport water could account for microbiota changes and appearance of *Aeromonas* spp. In order to validate this hypothesis both fish and fish transport water should have been analysed at the moment of introduction in the ornamental fountain L4, which was unfortunately not the case. 

**Table 1:** Antibiotic susceptibility of bacterial isolates identified from water in Lake (L1 and L2) and ornamental fountains (L3 and L4) in February.

ID	Bacteria	Antibiotic Susceptibility *						
		AMC	FOX	CAZ	CTX	IPM	GM	CIP
L1	*Klebsiella oxytoca*	R	S	S	S	S	S	S
	*Klebsiella pneumoniae*	R	S	S	S	S	S	S
	*Serratia marcescens*	R	S	S	S	S	S	S
	*Serratia odorifera*	R	S	S	S	S	S	S
	*Serratia rubidea*	S	S	S	S	S	S	S
	*Vibrio metschnikovii*	R	R	S	S	S	S	S
L2	*Elisabethkingia meningoseptica*	R	R	S	R	R	S	S
	*Enterobacter spp.*	R	S	S	S	S	S	S
	*Stenotrophomonas maltophilia*	R	R	R	R	R	S	S
L3	*Serratia rubidea*	R	S	S	S	S	S	S
L4	*Klebsiella pneumoniae ozonae 1*	S	S	S	S	S	S	S
	*Klebsiella pneumoniae ozonae 2*	R	S	S	S	S	S	S
	*Pastorella, Shigella*	S	S	S	S	S	S	S

* AMC: Amoxicillin/clavulanic acid, FOX: Cefoxitin (FOX), CAZ: Ceftazidime, CTX: Cefotaxime; IMP: Imipenem; GM: Gentamicin; CIP: Ciprofloxacin. R: Resistant, S: Susceptible; I: Intermediate.

**Table 2:** Antibiotic susceptibility of bacterial isolates identified from water in ornamental fountain (L4) in May.

Antibiotics	*A. Sobria*	*E. Aerogenes*
Amoxycillin	---	R
Amoxycilin/Clavulanic acid	---	R
Ampicillin	---	R
Ampicillin/Sulbactam	---	R
Piperacilin/Tazobactam	S	S
Cefepime	S	S
Cefotaxime	S	S
Cefoxitine	---	R
Ceftazidime	S	S
Cefuroxime	---	S
Ertapenem	---	S
Meropenem	S	S
Amikacin	S	S
Gentamicin	S	S
Ciprofloxacin	S	S
Levofloxacin	S	S
Fosfomycin	---	S
Nitrofurantoin	---	I
Trimetroprim/Sulfametoxazole	S	S

R: Resistant, S: Susceptible; I: Intermediate.

The organization of waterborne human pathogens within biofilms in this highly frequented area is a public health concern, so, we evaluated if clinically relevant bacteria recovered from biofilms were able to assemble biofilms *in vitro*. Also, this would be even more concerning if the bacteria prove antibiotic resistant. For this reason we evaluated the antibiotic susceptibility of the clinically relevant bacteria isolated from water and biofilms. The same antibiotic susceptibility profile was verified for *Aeromonas* isolates from water and biofilm samples. Nevertheless, *K. pneumoniae* isolates from biofilm samples exhibited resistance to cefoxitin in addition to amoxicillin/clavulanic acid resistance, when compared to water samples isolates. This observation mitigates the assumption that biofilm assembly is implicated in emergence of bacterial antibiotic resistance in general [21] and in particular *Klebsiella* spp., as we have recently reported [16]. It also showed that the synergism between antibiotic resistance and biofilm assembly is not exclusive of hospital and community aetiological agents, being extensive to environmental bacteria. Furthermore, the ability of all bacteria isolated from biofilms to assemble these structures *in vitro* was evaluated and the results are shown in Table 3. 

**Table 3:** Bacteria ability to assemble biofilms *in vitro* at different temperatures.

Biofilm Recovered Bacteria ID	OD 570 nm (AU)	
	25 °C	37 °C
*K. pneumoniae*	1.159 + 0.09	0.285 + 0.01
*A. sobria*	0.284 + 0.06	0.155 + 0.03
*A. veroni*	0.761 + 0.11	0.185 + 0.004
*C. violaceum*	0.017 + 0.01	0.096 + 0.05

**Figure 1:**
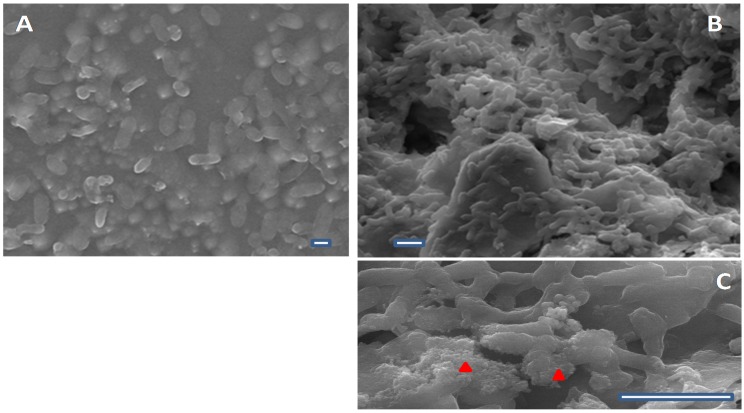
Biofilms assembled on cement at 25 °C (**A**) by *A. sobria* isolated from biofilms present in fountain L4 (May); (**B**) and *K. pneumoniae* isolated from biofilms present in lake L1 (February); (**C**) A detail of *K. pneumoniae* biofilm highlighting the extracellular matrix (red triangles) is shown. Scale bar 1 µm.

In all conditions *K. pneumoniae* is the best biofilm assembler and *C. violaceum* the worse. At 25 °C the *K. pneumoniae* showed 1.5- and 4-fold higher ability to form biofilms than *A. veroni* and *A. sobria*, respectively, and a 68-fold increase compared to *C. violaceum*. Figure 1 shows the differential biofilm-assembling performance of *A. sobria* (Figure 1A) and *K. pneumoniae* (Figure 1B,C) under conditions that mimic those found in the environment. The difference in biomass between the two *Aeromonas* species’ biofilms reaches 2.5-fold. The huge difference between the performance of *Aeromonas* and *C. violaceum* also suggest that the former could initiate biofilm assembly and the second joins it after, following kinetics similar to those described for oral biofilm formation [22]. Another observation emerged from analysis of these data is the better biofilm assembly performance was found at 25 °C (environmental temperature) than at 37 °C (human body temperature) for all bacteria except *C. violaceum*. This fact supports the idea that bacteria are able to adapt to distinct surrounding conditions.

## 4. Conclusions 

In the present study potential human pathogens were identified from samples of ornamental waters (fountains and lakes) from a typical urban park. These pathogens were present both in their planktonic form and organized within biofilms. Biofilm assembly by potential human waterborne pathogens represent a public health concern for easily accessible margins of either lakes or fountains located at highly frequented recreational areas. Waterborne pathogens such as *Aeromonas* species in biofilm or planktonic forms represent a significant cause of acute bacterial gastroenteritis in young children, with higher incidence in summer months. Although being a pilot study the obtained results support a periodic control of ornamental water microbiota as simple preventive measure to avoid potential health issues. 
